# 

**Published:** 1876-08

**Authors:** 


### TABLE OF CONTENTS.

ORIGINAL COMMUNICATIONS—

The Relation of Medical Editors to the Medical Profession in the United States, by A. N. Bell, M.D ........ 449

SELECTIONS—

Heat in the Treatmemt of Cerebro-Spinal Meningitis ........ 462
Local use of Bromide of Potassium. By Martin F. Coomes, M.D ........ 466
Illustrations of Heredity; Influence of Both Parents on the Children ........ 470
On the Treatment of the Diarrhœa of Typhoid Fever ........ 473

EXTRACTS AND GLEANINGS—

Cancers—477. Digitalis in Acute Diseases—481. Neurosis of the Joints and Their Treatment—483. Emulsions of Cod Liver Oil—484. The Treatment of Diarrhœa in Children—486. The Unmanageable Vomitings of Pregnancy—487. Hemiplegia; Use of Strychnia—Sore Nipples—Rheumatism—488. Diptheria—Radical Treatment of Prostatic Hypertrophy—489. Ergot in the Treatment of Galaetorrhœa and Mastitis—Calculus of the Bronchus—490. Local Application of Creosote in Hœmorrhages—Diptheria in the Adult Tracheotomy—Myelits a Sequel of Diarrhœa—491. Electrolysis in Nævi—Liquor Opii Sedativus—492. Leptandra as a Purgative—Dangers of Absorption of Carbolic Acid—493. On Bromide of Iron and its Preparation—494. Mania-a-potu—Hot Water Douche in Neuritis—494. Treatment of Severe Sprains—Guaiacum in Acute Diseases of the Throat—Mammitis and Mammary Abscess Treated by Bandaging—496. "No More Ovariotomy"—497. A New Antiseptic Dressing—498. Mistura Glycyrrhizæ Composita—Oleum Ovorum: or, Oil of Eggs—499. Boracic Acid as an Ordinary Dressing for Wounds—Perspiration of the Feet—Salicylic Acid—500. Cholbra Infantum—Crayons of Tannin—Leroy's Purgative Elixir—501. Pruritus in Pregnancy—Enuresis—Tapping the Gall Bladder—Resolvent Ointment—502. Stove Polish as an Irritant—Convulsions Arrested by the Sinistro-Lateral Posture—Hair Restorer—Aneurism Involving the Innominate, First Part of the Subclavian as well as Part of the Arch of the Aorta—503. Imperfect Mastication as a Cause of Diarrhœa—Cure for Warts—Cure for Corns—Harmless Cosmetic Powders—Chromic Acid for Warts—504. To Remove Stains Produced by the Nitrate of Silver—Fatal Poisoning by Alcohol—Gum Arabic Mucilage—Oil of Eggs for Sore Nipples, Chaps, etc.—505. Painful Menstruation—Lime Water in Infantile Eczema—Mode of Administering Creosote—Chlorate of Potassium in Chronic Gastro-Intestinal Catarrh—506. Pure English—Absorptive Power of Milk—Foot Powder—Erasive Soap to Remove Grease and Stains from Clothing—Cod-Liver Oil—507. A New Adhesive Plaster—Hypodermic Injection of Cold Water for the Relief of Pain—Remedy for Sick Headache—Camphorated Ether in Erysipelas—Dysentery—Wash for the Skin—508. Catarrh Snuff—Jalip Biscuit—Dentifrices—A Resolvent Ointment—Inspector-General H. Franklin, C. B—509.

EDITORIAL AND MISCELLANEOUS—

Our Advertising Department ........ 510
A Good Preparation ........ 510
Patents on Surgical Instruments ........ 510
Literary Notices ........ 511

